# Supplementary material for: Longitudinal Association between Stressful Life Events and Suicidal Ideation in Adults with Major Depression Disorder: The Mediating Effects of Insomnia Symptoms
Source: Behav Sci (Basel). 2024 May 31;14(6):467. doi: 10.3390/bs14060467 (PMC11200868; doi:10.3390/bs14060467)
Supplement: Supplementary file 1 [file behavsci-14-00467-s001.zip › behavsci-2942485-supplementary.pdf]

Supplementary Materials

In the Supplementary Material, Table S1 shows the result of sensitive analysis that compared the baseline sample characteristics between eligible and ineligible participants. Table S2 demonstrates the proportion of SLEs in persons with MDD. Figure S1 shows the mediating effects of insomnia symptoms on the relationship between different types of SLEs and SI.

Table S1. Baseline sample characteristics between eligible and ineligible participants.

Factor	Total, *n* (%)	Eligible	Ineligible	*P* value
Total (N, %)	1097(100.0)	511(46.6)	586(53.4)	NA
Gender (N, %)				
Male	325(29.6)	168(32.9)	157(26.8)	**0.033**
Female	772(70.4)	343(67.1)	429(73.2)	
Age (mean, SD)	29.4(6.9)	28.7(6.7)	30.1(7.0)	**0.001**
Marriage (N, %)				
Unmarried	795(72.5)	379(74.2)	416(71.0)	0.494
Married	280(25.5)	122(23.9)	158(27.0)	
Missing	22(2.0)	10(2.0)	12(2.0)	
Education level (N, %)				
Below undergraduate	472(43.0)	220(43.1)	252(43.0)	0.417
Undergraduate or above	623(56.8)	291(56.9)	332(56.7)	
Employed status (N, %)	2(0.2)	0(0.0)	2(0.3)	
Unemployed				
Employed	300(27.3)	158(30.9)	142(24.2)	**0.043**
Missing	794(72.4)	352(68.9)	442(75.4)	
Family monthly income (N, %)				
<10,000¥	391(35.6)	187(36.6)	204(34.8)	0.805
≥10,000¥	621(56.6)	286(56.0)	335(57.2)	
Missing	85(7.7)	38(7.4)	47(8.0)	
Smoking (N, %)				
No	606(55.2)	301(58.9)	305(52.0)	0.055
Yes	486(44.3)	207(40.5)	279(47.6)	
Missing	5(0.5)	3(0.6)	2(0.3)	
Drinking (N, %)				
No	155(14.1)	79(15.5)	76(13.0)	0.453
Yes	939(85.6)	431(84.3)	508(86.7)	
Missing	3(0.3)	1(0.2)	2(0.3)	
Exercise habit per week (N, %)				
No	701(63.9)	329(64.4)	372(63.5)	0.480
Yes	391(35.6)	181(35.4)	210(35.8)	
Missing	5(0.5)	1(0.2)	4(0.7)	
Moderate or severe depressive symptom (N, %)				
No	54(4.9)	29(5.7)	25(4.3)	0.349
Yes	1043(95.1)	482(94.3)	561(95.7)	
Moderate or severe anxiety symptom (N, %)				
No	215(19.6)	113(22.1)	102(17.4)	0.145
Yes	880(80.2)	397(77.7)	483(82.4)	
Missing	2(0.2)	1(0.2)	1(0.2)	
Resilience at baseline (N, %)				
Weak resilience (≤60)	1009(92.0)	471(92.2)	538(91.8)	0.630
Greater resilience	71(6.5)	34(6.7)	37(6.3)	
Missing	17(1.5)	6(1.2)	11(1.9)	
SLEs (N, %)				
No	384(35.0)	169(33.1)	215(36.7)	0.234
Yes	713(65.0)	342(66.9)	371(63.3)	

Table S1. Baseline sample characteristics between eligible and ineligible participants. (Continued)

Factor	Total, *n* (%)	Eligible	Ineligible	*P* value
SI at baseline (mean, SD)	3.9(2.6)	3.8(2.6)	4.1(2.6)	0.121
SI at baseline (N, %)				
No	224(20.4)	105(20.5)	119(20.3)	0.990
Yes	871(79.4)	405(79.3)	466(79.5)	
ISI scores (mean, SD)	15.1(6.4)	14.9(6.3)	15.3(6.6)	0.424

Note. SLEs= Stressful life events; SI= Suicidal ideation; ISI=Insomnia Severity Index.

Table S2. The proportion (%) of SLEs in persons with MDD (*n*=511).

**SLEs**	***n* (%)**
01. Life-threatening disease	33 (6.5)
02. Life-threatening accident	44 (8.6)
03. Physical assault	16 (3.1)
04. Bereavement	58(11.4)
05. Rape	12(2.3)
06. Other sexual assault	19(3.7)
07. Witnessed a traumatic event	76(14.9)
08. Childhood physical abuse	95(18.6)
09. Adulthood physical abuse	29(5.7)
10. Threatened	14(2.7)
11. Humiliated or discriminated	232(45.4)
12. Extreme fear or helplessness	215(42.1)

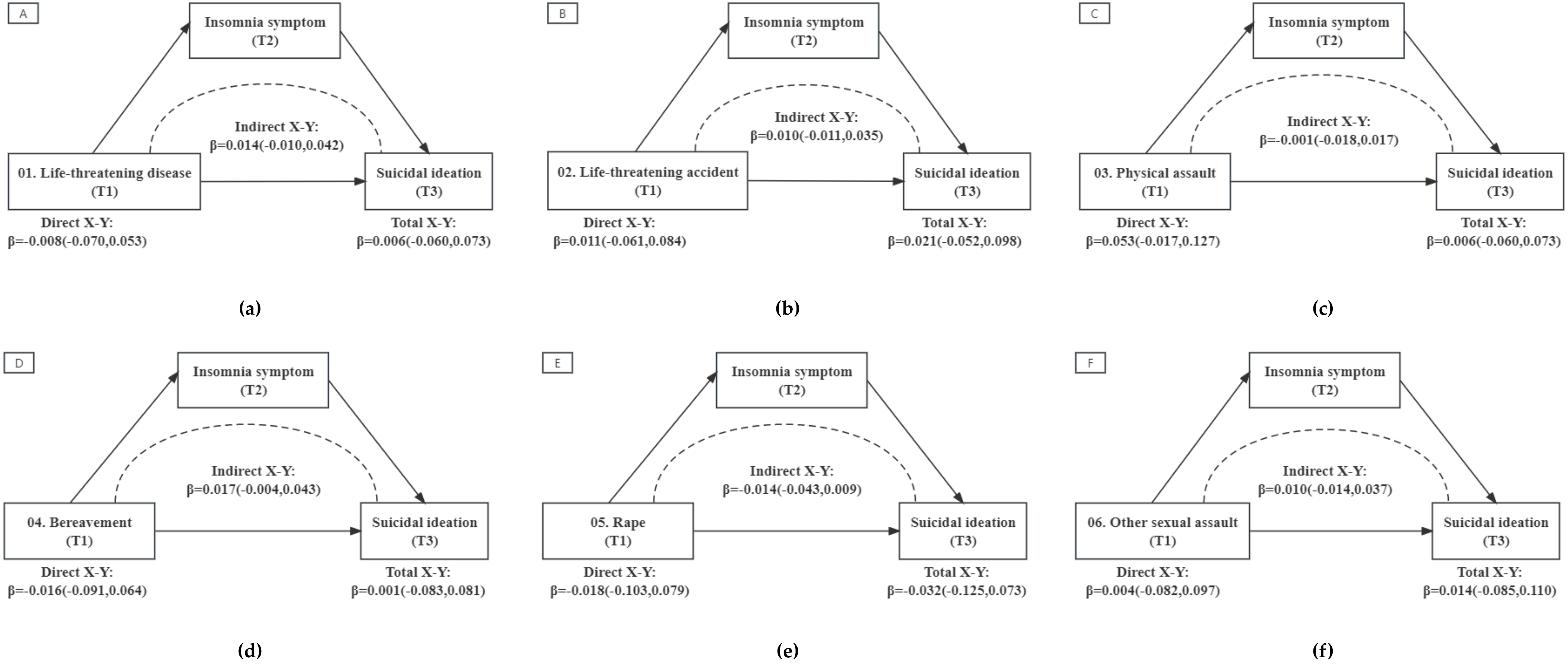

Figure S1. Mediating effects of insomnia symptoms on the relationship between different types of SLEs and SI.

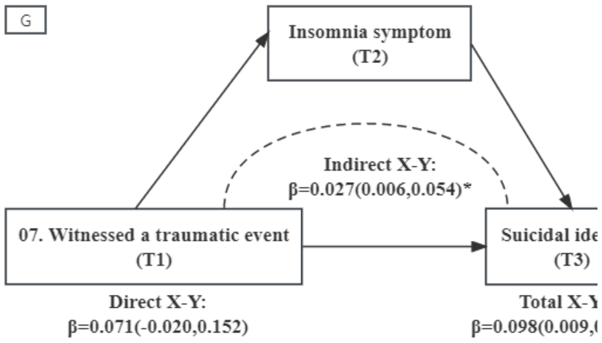

(g)

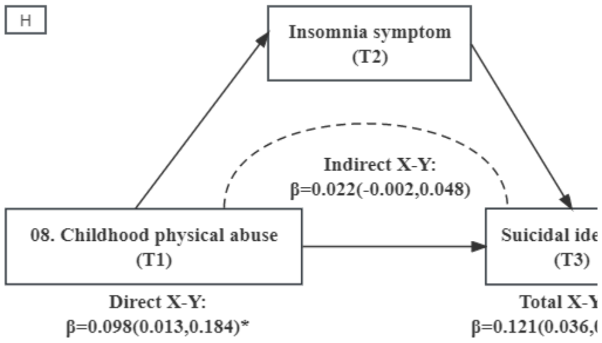

(h)

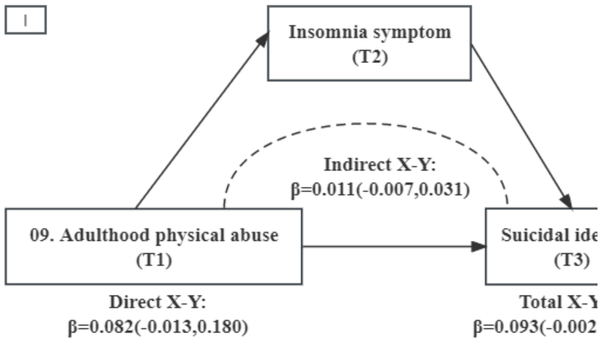

(i)

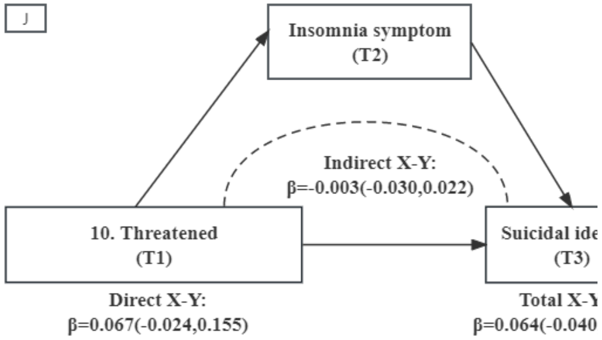

(j)

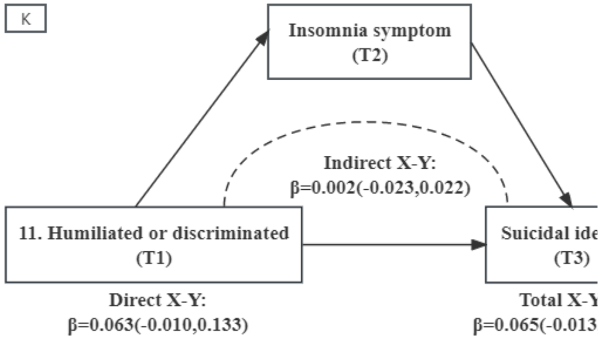

(k)

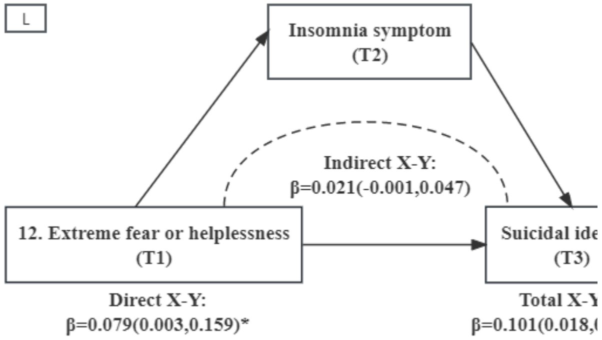

(l)

Figure S1. Mediating effects of insomnia symptoms on the relationship between different types of SLEs and SI (continued).

Note: *: *P* <0.05; **: *P* <0.01; T1=baseline; T2=12 weeks; T3=24 weeks. The structural equation models for SI were adjusted for gender, age, marriage, education level, employed status, family monthly income, smoking, drinking, exercise habit per week, depressive symptom, anxiety symptom, resilience, SI at baseline
